# Fanconi Anemia Proteins and Their Interacting Partners: A Molecular Puzzle

**DOI:** 10.1155/2012/425814

**Published:** 2012-03-29

**Authors:** Tagrid Kaddar, Madeleine Carreau

**Affiliations:** ^1^Department of Pediatrics, Université Laval, Cité Universitaire, Québec, QC, Canada G1K 7P4; ^2^Reproduction, Perinatal Health and Child Health, Centre de Recherche du CHUQ-CHUL, 2705 Boul Laurier, Québec, QC, Canada G1V 4G2

## Abstract

In recent years, Fanconi anemia (FA) has been the subject of intense investigations, primarily in the DNA repair research field. Many discoveries have led to the notion of a canonical pathway, termed the FA pathway, where all FA proteins function sequentially in different protein complexes to repair DNA cross-link damages. Although a detailed architecture of this DNA cross-link repair pathway is emerging, the question of how a defective DNA cross-link repair process translates into the disease phenotype is unresolved. Other areas of research including oxidative metabolism, cell cycle progression, apoptosis, and transcriptional regulation have been studied in the context of FA, and some of these areas were investigated before the fervent enthusiasm in the DNA repair field. These other molecular mechanisms may also play an important role in the pathogenesis of this disease. In addition, several FA-interacting proteins have been identified with roles in these “other” nonrepair molecular functions. Thus, the goal of this paper is to revisit old ideas and to discuss protein-protein interactions related to other FA-related molecular functions to try to give the reader a wider perspective of the FA molecular puzzle.

## 1. The FA Clinical Phenotype

Fanconi anemia (FA) is a complex disease that is considered a congenital form of aplastic anemia. The genetic mode of transmission is both autosomal and X-linked, and a growing number of identified genes are distributed among the various chromosomes. The common clinical manifestation in most patients with FA, which may occur in all FA patients eventually, is life-threatening bone marrow failure (BMF) [1, 2]. FA is also associated with diverse birth defects and a predisposition to malignancies. FA-associated congenital malformations can affect many organ systems including the central nervous system, the gastrointestinal system, and the skeletal system [3–8]. Other findings in patients with FA include short stature, skin pigmentation abnormalities, and small facial features. In addition, more than 70% of patients with FA show endocrine dysfunctions including deficiencies in growth hormone and thyroid hormone as well as diabetes [9, 10]. All of these disease manifestations suggest a role for FA genes in mechanisms that bear on hematopoiesis, development, and neoplasia.

## 2. The FA Molecular Pathway

Patients with FA are classified into complementation groups (to date 14 groups from A to P have been identified), and all of these groups correspond to one of the following cloned genes: *FANCA*, *FANCB*, *FANCC*, *FANCD1/BRCA2*, *FANCD2*, *FANCE*, *FANCF*, *FANCG*, *FANCI*, *FANCJ/BRIP1/BACH1*, *FANCL/PHF9, FANCM/HEF, FANCN/PALB2*,* and FANCP/SLX4 *[11–27]. Approximately 85% of FA patients have a defective FANCA, FANCC or FANCG gene, while the other genes account for less than 5% of the mutations found in FA patients. To date, some patients still remain unassigned indicating the possibility of novel FA genes [28]. Mutations in the *RAD51C* gene (provisionally termed *FANCO*) have been associated with a FA-like disorder, suggesting that this gene may represent yet another FA gene [29, 30]. Patients with mutations in one of the 15 FA and FA-like genes present clinical FA aspects to various degrees but show a common cellular phenotype: hypersensitivity to DNA cross-linking agents such as mitomycin C (MMC), diepoxybutane, and cisplatin [28, 31]. When exposed to those agents, cells from FA patients show an abnormally prolonged cell cycle arrest in the G2/M phase, increased chromosomal aberrations, and reduced survival. These cellular features define FA, and presumably, all FA proteins cooperate in a pathway, termed the FA pathway, to maintain chromosome integrity.

In the canonical FA pathway, FA proteins are subdivided into three complexes based on protein-protein interaction studies. The first complex, known as the core complex or complex I, is composed of seven FA proteins, including FANCA, FANCB, FANCC, FANCE, FANCF, FANCG, and FANCL [22, 27, 32–40]. The FANCM protein was also considered part of this core complex; however, further analysis revealed confounding results in FANCM-mutated cells [41]. Other proteins found in association with the core complex include Fanconi anemia-associated protein 24 (FAAP24), FAAP100, FANCM-associated histone fold protein 1 (MHF1), MHF2, and hairy enhancer of split 1 (HES1) [42–44]. All of these associated proteins are required for efficient FA pathway activation, but disease-causing mutations have yet to be found. Following DNA cross-link damage, the core complex associates with the FANCM-FAAP24 heterodimer on the chromatin [27, 39, 45, 46] and monoubiquitinates, through the activity of the FANCL E3 ubiquitin ligase, the complex II (or ID complex) components FANCD2 and FANCI [22, 47–50]. This ubiquitin tag alters the cellular distribution of complex II and promotes its association with the FA pathway complex III components FANCD1, FANCJ, FANCN and FANCP, and the FA-like disorder protein RAD51C [29, 30]. Complex III, similar to FANCM, is dispensable for the monoubiquitination of ID complex components, supporting the role of FA proteins in a linear (or canonical) response pathway. The deubiquitination of FANCD2 and FANCI by the UAF1/USP1 deubiquitinating enzyme complex appears to be required for the completion of the repair process [50–52].

## 3. FA Protein Cellular Localization

One intriguing aspect of the FA molecular pathway is the cellular distribution of FA proteins. Although the well-characterized function of this pathway in DNA crosslink damage occurs in the nucleus, FA core complex proteins can be found in different cellular compartments in addition to the nucleus.

 The first identified FA protein, FANCC, is principally found in the cytoplasm [53–56]. It was first reported that FANCC function in DNA cross-links resistance required its cytoplasmic location and that enforced nuclear FANCC expression abolished its ability to correct FA-C cells [55, 57]. It was later found that FANCC is partially localized to the nucleus, and this FANCC nuclear localization is critical for the function of the FA core complex in DNA crosslink responses [56, 58]. These conflicting results may suggest that FANCC is required in both cellular compartments, and that regulation of its protein level in each cellular compartment is important for its function. The molecular chaperone, glucose-related protein 94 (GRP94) involved in protein quality control, protein folding, and ER stress responses [59], directly binds FANCC and regulates its intracellular levels [60]. Reducing the levels of GRP94 through the use of a specific ribozyme affects FANCC stability and renders cells hypersensitive to MMC suggesting a possible mechanism of FANCC quality control and subsequent cellular localization.

FANCA cellular localization has been extensively studied [32, 36, 37, 61–63]. FANCA possesses a bipartite nuclear localization signal (NLS) domain that is required for its nuclear shuttling [64] and contains five nuclear export signal NES domains involved in chromosome region maintenance 1 (CRM1/exportin 1)-dependent nuclear export [65]. It has been proposed that the interaction of FANCA with the sorting nexin 5 protein (SNX5) may be involved in its subcellular trafficking [66]. Other studies have reported that FA core complex proteins could be found in the cytoplasm, in an unbound form, but also in a 600 kDa complex composed of at least four FA core complex proteins (i.e., FANCA, FANCC, FANCF, and FANCG) [57, 61, 67, 68]. Other studies have suggested the formation of different cytoplasmic subcomplexes, including FANCA/FANCG [36, 38], FANCB/FANCL [23], and FANCC/FANCE [33, 69–71]. It has been suggested that these subcomplexes are probably translocated to the nucleus independently before association into a core complex through FANCF, which acts as a linker protein [33, 72].

Because the nuclear presence of both FANCC and FANCE depends on each other, an interdependence between FA proteins has been proposed. Indeed, studies on FA mutant cell lines have shown that the core complex fails to form if one protein is absent or mutated [33]. Studies of synchronized cells have shown that FA proteins shuttle between the nucleus and the cytoplasm during the cell cycle. Early in the G1 phase, FA core complex proteins are localized to the cytoplasm, at the G1-S border, they are loaded onto chromatin, and throughout mitosis they migrate to the nuclear periphery to become completely excluded from the condensed chromosomes [67, 73, 74]. The exclusion of FA proteins from condensed chromosomes occurs in the absence of DNA damage, whereas treatment with MMC results in an increased binding of core complex proteins to chromatin [67, 73, 75]. Considering these results, it is not inconceivable to surmise that the cytoplasmic forms of FA proteins or FA subcomplexes are likely to be critical for cell signaling events in normative conditions.

## 4. Posttranslational Modification of FA Proteins

FA proteins undergo multiple posttranslational modifications, including monoubiquitination, phosphorylation and proteolytic processing. The most widely studied modification is the monoubiquitination of FANCD2 and FANCI. Although only two of the fifteen FA proteins are monoubiquitinated, several FA proteins, including FANCA, FANCE, FANCG, FANCD2, FANCI, and FANCM, are phosphorylated, and two FA proteins are regulated through a caspase-mediated proteolytic process. These data suggest that posttranslational modifications play an important role in FA proteins activity. In this section, we will only discuss FA protein modifications other than the ubiquitination of FANCD2 and FANCI because it has been extensively discussed [47, 76, 77].

The FANCA protein was the first FA protein shown to be phosphorylated [58, 63, 78]. The FANCA phosphorylation site was first thought to be located at S1149 [79], which harbors an AKT kinase consensus sequence; however, the FANCA S1149A mutant was shown to be more efficiently phosphorylated than wildtype FANCA. The FANCA phosphorylation site was later identified by mass spectrometry as serine 1449 [80]. The phosphorylation of FANCA at S1449 is functionally important because it was found to be defective in lymphoblasts from several patients with FA, and FANCA S1449A failed to fully rescue FA-A mutant cells. A novel wortmannin-sensitive protein kinase termed FANCA-PK was first suggested as the kinase responsible for FANCA phosphorylation [81]; however, the true kinase that phosphorylates FANCA in response to DNA damage was later identified as ATR [80]. FANCA was also shown to associate with the IKK signalosome through direct interaction with IKK2, the I*κ*B Kinase-2 [82]. Although this kinase affected the phosphorylation state of several FANCA-associated proteins, no clear, direct phosphorylation of FANCA by IKK2 has been reported. Different groups have reported that FANCG is also phosphorylated. FANCG phosphorylation occurs at serines 7, 383, and 387, with the two latter sites being cell cycle-dependent and actively phosphorylated during mitosis [83–85]. The exclusion of FA proteins from chromosomes during mitosis appears to coincide with phosphorylated FANCG. All the FANCG phosphorylation sites are functionally important because mutant forms of these FANCG serine residues compromised its ability to rescue FA-G mutant cells. The FANCC-interacting protein kinase cdc2 [86] was shown to be required for the phosphorylation of at least the S387 FANCG residue. The kinases responsible for the phosphorylation of the other FANCG target sites are unknown.

The FANCD2 protein undergoes two different posttranslational modifications. In addition to monoubiquitination on lysine 561, FANCD2 is phosphorylated on several residues by different kinases depending on the cellular signal. In response to various DNA damaging agents, including ultraviolet light, MMC, hydroxyurea, and ionizing radiation, FANCD2 is phosphorylated on T691 and S717 followed by S222 in an ATM/ATR-dependent mechanism [87, 88]. S222 phosphorylation triggers the activation of an intra-S-phase checkpoint response. In addition, the FANCD2-T691A/S717A double mutant does not complement the MMC sensitivity in FA-D2 cells, suggesting that this posttranslational modification is required for FANCD2 function in the DNA damage response. Other ATM-dependent FANCD2 phosphorylation sites have been described and have been shown to be functional in *in vitro* assays, including S1401, S1404, and S1418, and only S1401 has been confirmed *in vivo*. S331 of FANCD2 has been shown to be phosphorylated by the checkpoint kinase 1 (CHK1) and is essential for MMC resistance [89]. Although FANCD2 phosphorylation is independent of other posttranslational modifications, it promotes or enhances the monoubiquitination process. For instance, ATR-mediated phosphorylation of FANCD2 is essential for its monoubiquitination in response to DNA damage as shown by absence of FANCD2 monoubiquitination in ATR-deficient cells and cells from patients with Seckel syndrome, a disease resembling FA [90]. FANCI was identified in the search for ATR-inducible phosphorylated proteins in response to ionizing radiation [50]. Three phosphorylation sites were detected in the human FANCI protein (S730, T952, and S1121), and two other sites were detected in the mouse protein (S555 and T558). FANCI phosphorylation is essential for the FA pathway activation following DNA damage as measured by FANCD2 monoubiquitination [91].

FANCE and FANCM phosphorylation has also been studied in the context of induced DNA damage. In response to DNA damage, CHK1 was shown to phosphorylate FANCE on two residues, notably T346 and S374 [92]. FANCE phosphorylation is required for MMC resistance but is dispensable for FA pathway activation as measured by FANCD2 monoubiquitination. The CHK1-induced phosphorylation of FANCE promotes its assembly with FANCD2 into nuclear foci and promotes its degradation, serving as a potential negative regulatory mechanism of the FA pathway. FANCM is a phosphoprotein that contains multiple predicted ATR phosphorylation sites and becomes hyperphosphorylated in response to DNA damage and during mitosis [27, 93]. FANCM phosphorylation occurs independently of the FA core complex activation leading to the monoubiquitination of the ID complex.

Finally, another posttranslational modification found in FANCC is caspase-mediated proteolytic processing [94]. Similar to many proteins involved in signaling mechanisms, FANCC is cleaved by a caspase during apoptosis. This proteolytic modification of FANCC is not required for its function in DNA repair or DNA damage signaling, but the cleavage of FANCC inhibits its suppressor of apoptosis function. Recently, a second FA protein, FANCD2 was shown to be regulated by a caspase-mediated proteolytic processing in response to DNA crosslink damage and the non-DNA damaging agent TNF-*α*  [95]. Both the TNF-*α*  and DNA crosslink-mediated disappearance of FANCD2 was blocked by caspase inhibitors but not by proteasome inhibitors, suggesting that FANCD2 is regulated through a caspase-dependent mechanism in response to cellular stress.

## 5. FA Proteins Partners with Roles in Oxidative Metabolism

The abnormal sensitivity of FA cells to reactive oxygen species (ROS) was first suggested in 1977 by Nordenson [96] who showed reduced chromosomal breaks in FA lymphocytes cultured in the presence of superoxide dismutase, catalase, or both enzymes. The role of oxygen on chromosomal instability in FA mutant cells was confirmed by Joenje et al. in 1981; they showed attenuated chromosomal aberrations at low oxygen tension (5%) but aggravated chromosomal aberrations at high concentrations of oxygen [97]. Subsequently, several reports indicated that FA cells were hypersensitive to oxygen radicals showing reduced growth and blockage in the G2 phase of the cell cycle [98–101]. Increased ROS in FA leucocytes has also been reported [99]. In addition, the overexpression of detoxifying enzymes, the inhibition of enzymes involved in oxidation or the use of antioxidants in FA cells reduced the rate of spontaneous chromosomal breakage and abolished the DNA damaging effects of MMC [98, 102–106]. Other studies have established a link between an altered redox state and reduced proliferation, reduced growth, and altered cytokine responses in FA cells including hematopoietic progenitors [107–110]. Studies from FancC/Sod1 double mutant mice exhibiting defects in hematopoiesis including bone marrow hypocellularity and cytopenia, which is reminiscent of phenotypes observed in patients with FA, suggest that an abnormal redox state contribute to BMF in FA [111].

Together, these data indicate that FA proteins may be involved in responses to endogenous oxidative stress or in the regulation of the cell redox state. This hypothesis is further supported by studies showing interactions between FA proteins and proteins involved in oxygen metabolism [112]. For instance, FANCC has protein partners with roles in redox metabolisms, including glutathione *S*-transferase  *π*  I (GST*π*I) and NADPH cytochrome-P450 reductase (RED) [113, 114], whereas FANCG interacts with cytochrome P450 2E1 (CYP2E1) and the mitochondrial peroxiredoxin-3 (PRDX3) [68, 112, 115]. Through these interactions, FA proteins were shown to attenuate the redox activation of xenobiotics and to prevent apoptosis. Consequently, in FA mutant cells, a loss of interaction between FA proteins and these molecular antioxidants leads to an aberrant redox metabolism that translates into ROS-mediated DNA damage and cell death (see Table 1).

## 6. FA Proteins Partners with Roles in Cytokine Signaling and Apoptosis

It is well established that FA mutant cells are prone to apoptosis. The FA literature is rich in reports pertaining to FA mutant cells (human bone-marrow-derived cells, lymphocytes, fibroblasts, and mouse embryonic fibroblasts) that show increased apoptosis or reduced cell growth in response to various agents including ROS inducers, DNA damaging agents, growth factor withdrawal, and cytokines. It is clear from many studies of patient-derived cells and cells from FA mouse models that FA proteins are involved in pathways that regulate cell survival or cell death [116–121]. For instance, two FA proteins, FANCC and FANCD2, are caspase targets [94, 95], and FANCC overexpression or the inhibition of its caspase-mediated cleavage prevents or delays apoptosis, even in wildtype cells supporting the idea of a cell survival function of the FA proteins [94, 119, 122]. The role of FANCC in cell survival has been linked to oxidative metabolism as described above but it may also be linked to cytokine-mediated cellular responses because many cytokine-mediated signaling events lead to apoptosis. It has been suggested that abnormal cytokine regulation may account for the progressive BMF observed in patients with FA because TNF-*α*  overproduction and underproduction of Il-6 have been detected in the sera of patients with FA [123–125]. FA-C mutant cells and *FancC^−/−^* progenitor and stem cells are hypersensitive to the inhibitory cytokines including TNF-*α*  and IFN-*γ*, and show suppressed growth and increased apoptosis at doses that do not affect normal cells [116, 122, 126, 127]. In addition, the continuous injection of low IFN-*γ*  doses *in vivo* leads to BMF in FA mice [128, 129], whereas TNF-*α*  leads to clonal evolution and leukemia in this FA mouse model [130]. In support of these altered cytokine responses in FA cells, the cytokine-response genes myxovirus A (*MxA*), IFN response factor 1 (*IRF1*), *p21^CIP/WAF^*, and IFN-stimulated gene factor 3 (*ISGF3*γ**) were highly expressed in FA mutant cells without exogenous cytokine stimulation, while corrected cells suppressed this overproduction and restored their MMC resistance [116, 131, 132]. These data suggest that FA proteins, or at least FANCC, function to modulate a cytokine-mediated signal. Indeed, FANCC was shown to directly interact with signal transducer and activator of transcription 1 (STAT1), which is an IFN signal transducer [133]. FANCC functions as a control factor for STAT1 docking at the IFN-*γ*R complex and subsequent activation of the IFN type II signaling cascade [133]. Thus, the STAT1 activation defect observed in FA-C cells results in an altered nuclear STAT1-DNA complex, which diminishes the expression of IRF1. The STAT1-FANCC interaction is also induced by other cytokines, including IFN-*α*, granulocyte-macrophage colony-stimulating factor (GM-CSF), and stem cell factor, whereas mutant FANCC does not associate with STAT1 in cells stimulated with these factors. FANCC seems to regulate IFN*γ*-inducible genes (e.g., IRF1, p21^WAF^, and ISGF3*γ*) independently of STAT1 binding. An altered response to type I IFN was also observed in FANCC mutant and *FancC^−/−^* cells, as shown by the reduced phosphorylation of the Janus kinases, Jak1 and Tyk2, and the subsequently diminished phosphorylation of STAT1, STAT3, and STAT5 [134]. This altered Tyk2 response translates into reduced numbers of CD4-positive cells in *FancC^−/−^* mice. Because Tyk2 plays a role in the differentiation and maintenance of T helper cells, failure of FANCC to normally activate Jak/STAT signaling may result in impaired immune cell differentiation and immune defects, as reported in patients with FA [135–139].

FANCC has been shown to physically interact with Hsp70 [140]. This interaction appears to be required for protection against TNF-*α*  and IFN-*γ*-induced apoptosis because reduced Hsp70 expression sensitizes normal cells to these cytokines but does not further augment the hypersensitivity to apoptosis in FA-C cells. Because Hsp70 is known to suppress the IFN-inducible double-stranded RNA (dsRNA)-dependent protein kinase (PKR) activation [141] and FA-C cells have constitutively activated PKR [142], FANCC was shown to inhibit the kinase activity of PKR through physical interaction with Hsp70 [143]. Although this activity is independent of a functional FA complex, the FA core complex protein FANCA was found to interact with IKK2 (or IKK*β*) a kinase and a component of the IKK signalosome [82]. The IKK signalosome is a critical mediator of the cellular response to stressors such as dsRNA and cytokines [144, 145]. Deletion of IKK2 has been shown to affect the development of CD4-positive cells [146]. Because *FancC^−/−^* mice have reduced numbers of CD4^+^ cells and two FA proteins have partners that participate in cytokine-activated signaling cascades affecting the development of these lymphocytes, we can speculate that FA proteins may act as converging key molecules.

## 7. FA Protein Partners with Roles in Transcription

Another FA protein role less considered is the regulation of transcription. Several FA proteins have interacting partners directly involved in transcriptional regulation. The first FA protein partner identified that acts in transcription is FAZF (FA Zinc Finger) [147]. FAZF, also known as RoG (for repressor of GATA) [148], PLZP (for PLZF-like zinc finger protein) [149] and TZFP (for testis zinc finger) [150], is a transcriptional repressor that belongs to the BTB/POZ family of proteins and is similar to the PLZF protein [147]. This family of transcriptional repressors was shown to be important for several developmental processes including tissue proliferation and differentiation and tumor formation. FAZF was identified in a yeast 2-hybrid screen with FANCC. FAZF was shown to be highly expressed in CD34-positive progenitor cells; it further increased during proliferation of these cells and decreased during their terminal differentiation [151]. FAZF acts as a negative regulator of transcription. Because a disease-causing mutation in FANCC interferes with FAZF binding [147], and *FancC^−/−^* hematopoietic stem/progenitor cells show increased cycling and aberrant cell cycle control [152], a plausible hypothesis is that the FANCC-FAZF interaction in hematopoietic stem/progenitor cells leads to the repression of critical target genes required for growth suppression.

A second transcriptional repressor identified as a FA-binding protein is the hairy enhancer of split 1 (HES1) [44]. HES1 is a member of a highly conserved family of hairy-related basic helix-loop-helix (bHLH)-type transcriptional repressors. HES1 was shown to interact directly with several components of the FA core complex. The FA core complex was shown to contribute to the transcriptional regulation of HES1-responsive genes, both positively (*HES1*) and negatively (cyclin-dependent kinase inhibitor *p21^cip1/waf1^*). Two mechanisms of regulation by FA proteins have been proposed. The first proposed mechanisms is that by interacting with HES1, FA core complex proteins antagonize HES1-mediated transcriptional repression by interfering with the assembly of the HES1/transducing-like-enhancer of split (TLE) corepressor complex at the *HES1* promoter [153]. The second proposed mechanism involves an indirect mechanism where the binding of FA proteins with HES1 influences HES1 affinity or its specificity for promoters, such as that of *p21^cip1/waf1^*.

The brahma-related gene 1 protein (BRG1) has also been identified as a FA-binding partner through a yeast 2-hybrid screen [154]. BRG1 is the central catalytic subunit of the SWI/SNF family of ATP-dependent chromatin remodeling complexes [155]. BRG1 is a major coregulator of transcription, both in activation and repression, through chromatin modulation. Although the FANCA-BRG1 interaction has been shown in cells, the functional impact of this interaction remains unclear.

In view of these FA protein partners with roles in transcriptional regulation and the fact that the FA core complex possesses E3 ubiquitin ligase activity, it is possible that FA proteins act as transcriptional coregulators through the posttranslational modification of these transcriptional regulators.

## 8. Conclusion

**Figure 1:**
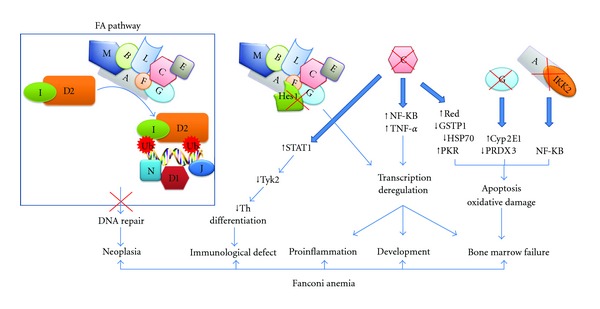
Putative roles of FA proteins through their interacting partners. The involvement of FA proteins with their protein partners in the different molecular mechanisms that lead to regulation of transcription, cell cycle regulation, ROS detoxification, DNA repair, and cell survival. Loss of protein interactions between FA proteins and their partners through disease causing mutations in a FA gene could lead to a defective molecular function resulting in an array of phenotypes including BMF and congenital malformations.

Since the discovery of *FANCC*, the first identified FA gene in 1992 [15], there have been significant advances in the FA molecular biology field. These advances mostly include characterization of the canonical FA pathway, which is activated in response to DNA crosslink damage. It is clear that FA proteins are required for DNA crosslink repair; however, the question of how a defective FA protein leads to BMF, and developmental abnormalities remains elusive. It is obvious that absence of a functional FA protein affects many cellular and molecular functions and leads to an array of cellular phenotypes (see Figure 1). A perplexing question is whether FA proteins interactions with their nonrepair partners act only as modifiers of the clinical manifestation of FA. Once we reconcile all the notions related to FA proteins role in these various cellular and molecular activities, we may then obtain a clearer picture of the complexities of this molecular puzzle.

## Figures and Tables

**Table 1:** List of FA protein partners.

Functional class	Specific function	Protein name	Interacts with	References
Transcription	Transcriptional repressor	FAZF	FANCC	[112, 147]
		HES1	FANCA, F, G, L	[44]
	Stress-induced chaperone	Hsp70	FANCC	[112, 140]
		GRP94	FANCC	[60, 112]
	Chromatin modifier	BRG1	FANCA	[112, 154]

Cell cycle	Serine/threonine kinase	cdc2	FANCC	[86, 112]

Cell signaling	Cytokine response	STAT1	FANCC	[133]
		IKK2	FANCA	[79, 82, 112]
	Secondary modification	Akt kinase	FANCA	[79, 112]

Oxidative metabolism	Electron transfer	RED	FANCC	[112, 114]
	Cytosolic Detoxifying enzyme	GSTP1	FANCC	[112, 113]
	Metabolism of xenobiotics	CYP2E1	FANCG	[112, 115]
	Antioxidant enzyme	PRDX3	FANCG	[68]

Transporter	Intracellular trafficking	SNX5	FANCA	[66, 112]
